# Microresonator photonic wire bond integration for Kerr-microcomb generation

**DOI:** 10.1038/s41598-024-79945-4

**Published:** 2024-11-23

**Authors:** Alain Yuji Takabayashi, Nikolay Pavlov, Victoria Rosborough, Galen Hoffman, Lou Kanger, Farzad Mokhtari Koushyar, Taran Huffman, Mike Nelson, Charles Turner, Leif Johansson, Juergen Musolf, Henry Garrett, Thomas Liu, Gordon Morrison, Yanne Chembo, Brian Mattis, Thien-An Nguyen, Mackenzie Van Camp, Steven Eugene Turner, Maxim Karpov, John Jost, Zakary Burkley

**Affiliations:** 1 Enlightra, Rue de Lausanne 64, 1020 Renens, VD Switzerland; 2 Freedom Photonics, 41 Aero Camino, Santa Barbara, CA 93117 USA; 3 GXC, 10000 Metric Blvd., Austin, TX 78758 USA; 4 FAST Labs™, BAE Systems, 130 Daniel Webster Hwy., Merrimack, 03054 NH USA; 5 University of Maryland, 8279 Paint Branch Dr., College Park, MD 20742 USA; 6 ORCA Computing, 10000 Metric Blvd., Austin, TX 78758 USA

**Keywords:** Microresonators, Solitons, Nonlinear optics, Optics and photonics, Integrated optics, Microwave photonics

## Abstract

Extremely high-*Q* microresonators provide an attractive platform for a plethora of photonic applications including optical frequency combs, high-precision metrology, telecommunication, microwave generation, narrow linewidth lasers, and stable frequency references. Moreover, the desire for compactness and a low power threshold for nonlinear phenomena have spurred investigation into integrated and scalable solutions. Historically, crystalline microresonators with *Q* $\sim$ 10^9^ were one of the first material platforms providing unprecedented optical performance in a small form factor. A key challenge, though, with these devices is in finding alternatives to fragile, bulky, and free-space couplers, such as tapered fibers, prisms, and cleaved fibers. Here, we present for the first time, the evanescent coupling of a photonic wire bond (PWB) to a MgF_2_-based microresonator to generate solitons and a pure, low-noise microwave signal based on Kerr-microcombs. These results open a path towards scalable integration of crystalline microresonators with integrated photonics. Moreover, because PWBs possess advantages over traditional coupling elements in terms of ease of fabrication, size, and flexibility, they constitute a more advanced optical interface for linear and nonlinear photonics.

## Introduction

High-*Q* optical microresonators present unique properties in the various fields of modern photonics. Their small size and high optical field density provide an opportunity to generate various nonlinear effects at low input optical power and low power consumption within a scalable and compact form factor. The generation of optical Kerr frequency combs in microresonators^1^ has led to an even wider application range^2^ and there are currently three primary platforms for high-*Q* microresonators. These categories include microresonators made in silica^3,4^, alkaline earth fluorides^5–7^, otherwise referred to as crystals, and photonic integrated circuits (PICs). Both crystalline and PIC-based platforms have achieved remarkable milestones, including Dissipative Kerr microcomb generation^1,8^, pure microwave signal generation^9–11^, dual-comb sources^12–14^, high-capacity telecommunication^15–18^, on-chip atomic clocks^19^, calibration of spectrometric equipment^20,21^, optical beam-steering for LiDAR^22,23^, and narrowline lasers and microcombs based on the self-injection locking effect^24–27^. A surge of interest in compact, ultra-low-noise microwave signal generation using microcombs, in particular, can be traced to recent publications on chip-based^28–30^, as well as MgF_2_ crystalline resonator-based^31^ microwave oscillators.

Bulk crystalline microresonators often offer superior performance by virtue of their exceptionally high-quality factors. These devices are mass-manufactured from a variety of materials^32,33^ and support ultra-high quality factors ($\sim$ 10^9^) within the ultraviolet to mid-infrared wavelength range^34–37^. Of these materials, MgF_2_ naturally exhibits anomalous material dispersion in the C-band enabling microcomb generation without the need for the complicated geometries associated with dispersion engineering^1,9,25^. The typical soliton repetition rate for MgF_2_ microresonators is tens of GHz, and due to their extremely high-*Q*, the nonlinear power threshold is in the milliwatt regime^9^. Low repetition rates in the GHz regime are compatible with standard microwave electronics and as such can be directly accessed without need for additional frequency down-conversion architectures. PIC-based microcombs, on the other hand, require power levels at the watt-level for solitons with these repetition rates^11^. Additionally, the larger effective mode area (volume) of crystalline microresonators, as compared to their PIC-based equivalents, leads to lower thermorefractive noise (TRN) and hence, improved laser frequency stabilization. TRN reduction in PICs on the other hand, requires design adaptations and additional fabrication steps^38,39^.

**Figure 1:**
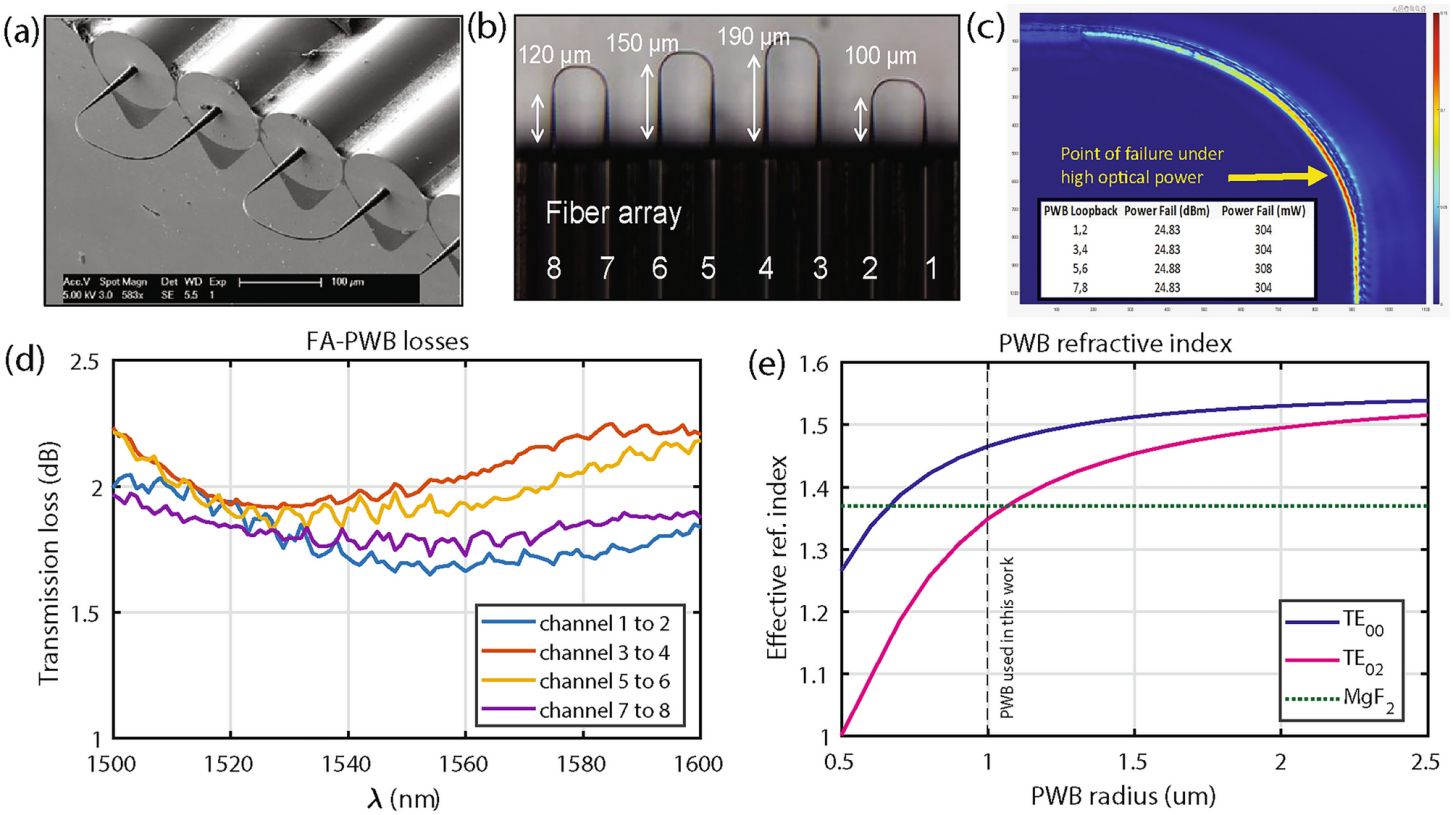
Design and characterization of PWBs. **(a)** SEM micrograph of the PWBs written to the fiber cores of an eight-channel V-groove fiber array. **(b)** Microscope image showing four PWBs, each with a different extension length: 100 µm, 120  µm, 150 µm and 190 µm respectively. **(c)** Sub-micron-resolution thermo-reflectance image of a PWB indicating optical power concentration within the loopback. The insert table shows the optical power of PWB failure. **(d)** Fiber-to-fiber losses through the PWBs in (**b**) indicating losses $\sim$ 1.7 dB at 1550 nm for the shortest PWB (blue curve 100 µm length). **(e)** Finite-element method simulation on the effective index of two TE modes (TE$_{00}$ is the blue curve, TE$_{02}$ is the pink curve) within the PWB as a function of cross-sectional geometry (PWB radius) to reach index-matching with MgF_2_ (green dashed line) at 1550 nm.

**Figure 2:**
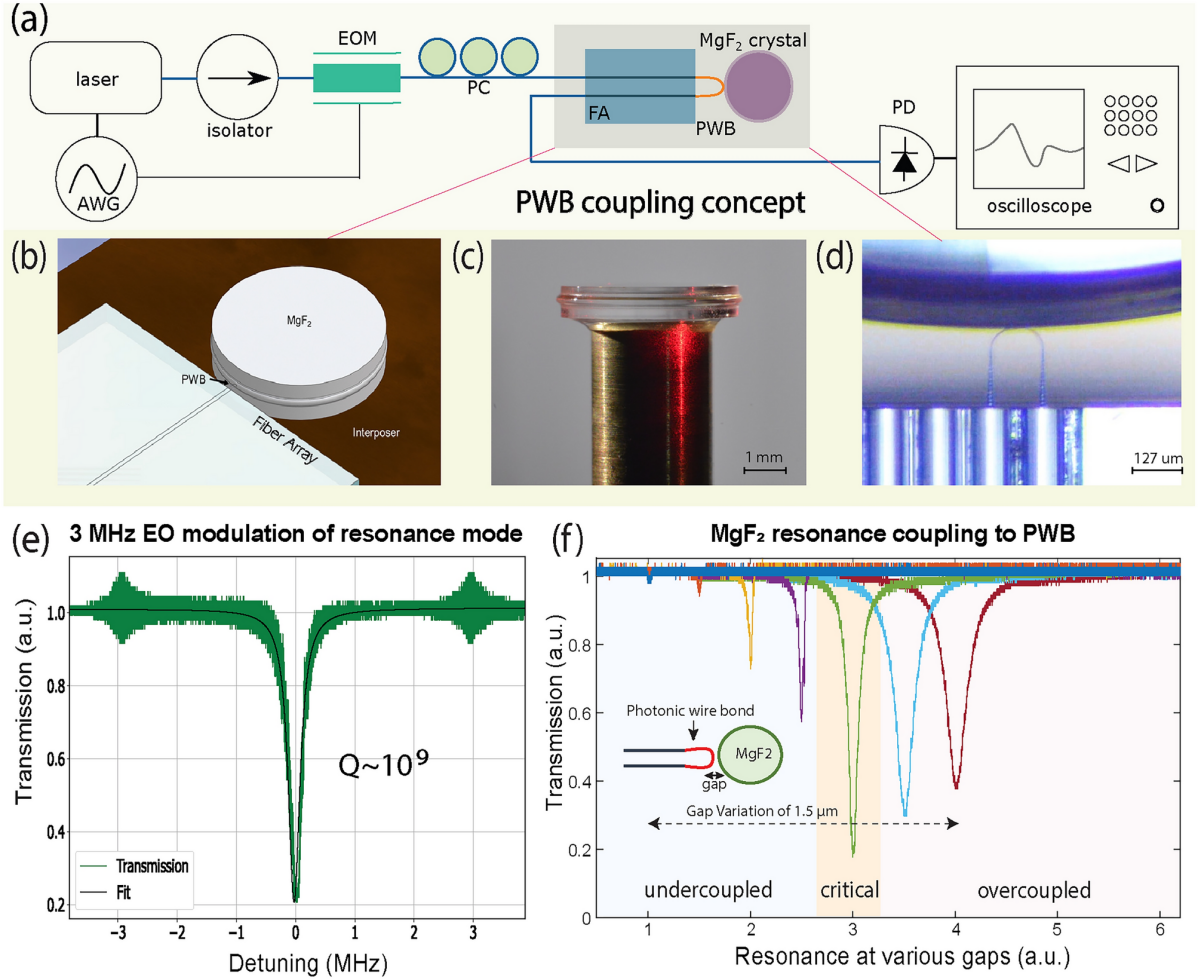
PWB-microresonator characterization in linear regime. **(a)** Experimental setup for testing the PWB-microresonator configuration in the linear regime, i.e., coupling ideality and *Q*-factor measurement. *AWG* arbitrary waveform generator, *EOM* electro-optical modulator, *PC* polarization controller, *FA* fiber, *V* groove array, *PWB* photonic wire bond, *PD* photodetector. **(b)** 3D rendering of the FA-PWB-crystal arrangement. **(c)** Photograph of a 4.92 mm MgF_2_ crystal with microresonator protrusion. **(d)** Microscope image of PWB on the FA facet coupled to the crystalline microresonator. **(e)** Linewidth measurement of a resonance with 3 MHz calibration sidebands, wherein a Lorentzian fit (black line) has been applied to the measured transmitted light (green line) and gives a linewidth of 240 kHz, corresponding to a *Q*-factor of $\sim$ 10^9^ at 1550 nm. **(f)** Evolution of resonance linewidth as a function of PWB-microresonator gap for a single resonance. All traces correspond to the same resonance and have been offset to better visualize the transition from undercoupled (left), to critically coupled (center), to overcoupled (right) states. Furthermore, the total displacement between undercoupled to overcoupled regimes is 1.5 $\upmu$m, starting from 0 $\upmu$m on the left.

**Figure 3:**
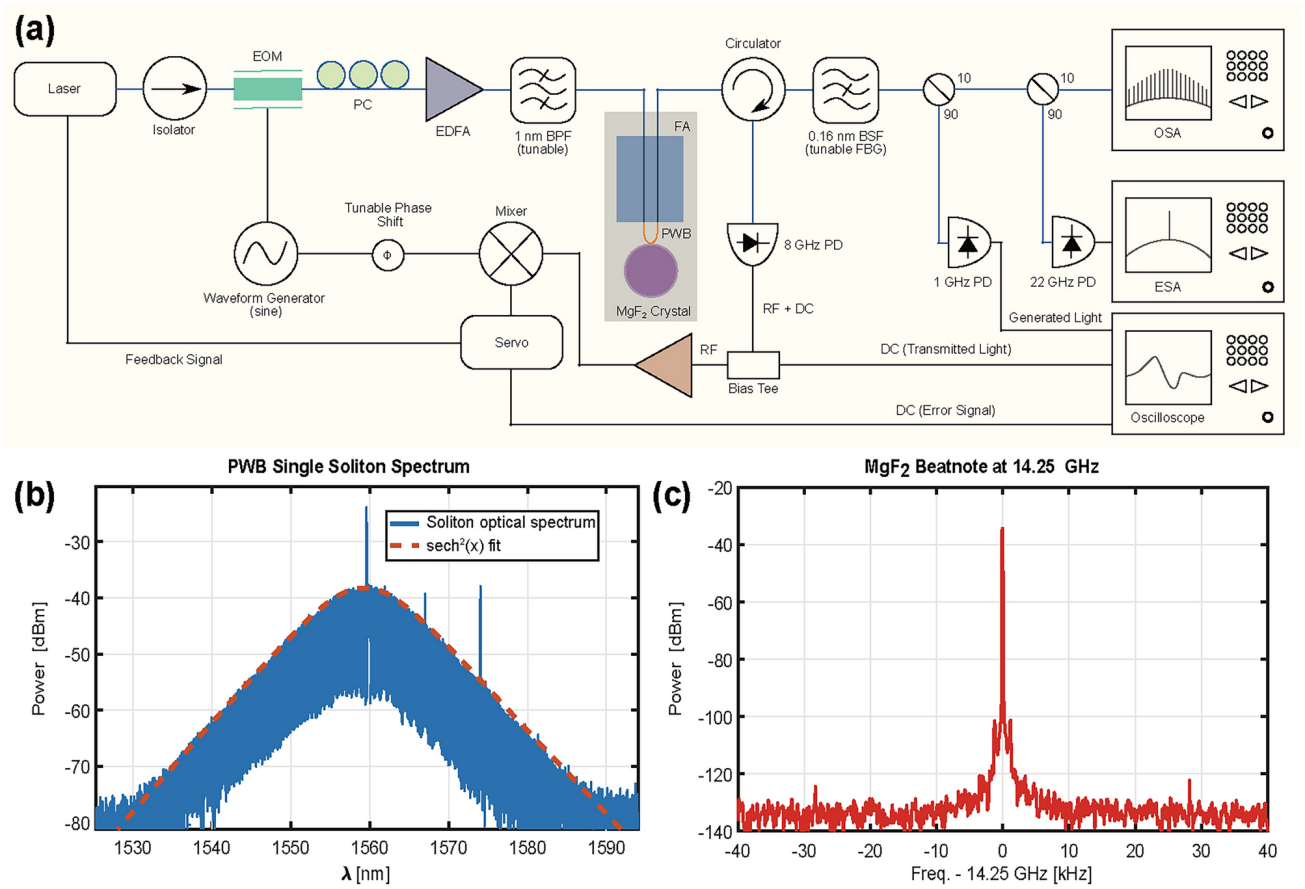
PWB-microresonator characterization in nonlinear regime. **(a)** Experimental setup used in the nonlinear characterization of the PWB-microresonator system. *EOM* electro-optical modulator, *PC* polarization controller, *EDFA* erbium-doped fiber amplifier, *BPF* tunable bandpass filter, *FA* fiber, *V* groove array, *PWB* photonic wire bond, *FBG* tunable fiber Bragg grating notch filter, *PD* photodetector, *OSA* optical spectrum analyzer, *ESA* electrical spectrum analyzer, *Servo* PID servo controller. **(b)** A single soliton optical spectrum generated in a MgF_2_ microresonator with a fitted $sech^2(x)$ envelope shown by the dashed red line. **(c)** ESA trace at a 10 Hz resolution bandwidth (RBW) for an 80 kHz span of the 14.25 GHz beatnote signal, which corresponds to the comb line spacing in the soliton state.

Although crystalline microresonators possess key advantages over PIC-based microresonators, such as lower threshold powers for nonlinear phenomena and simpler design and fabrication, the primary challenge has been in optical coupling. Conventional approaches include tapered optical fibers^40^, prisms^41^, angle-cleaved fibers^42^, and grating-based fiber couplers^43^. These approaches can be bulky, fragile or require free-space optical elements and thus do not readily support high-volume production, as would be required for crystalline microresonators to compete with PIC-based solutions. One notably space-efficient demonstration uses a free-hanging silica waveguide on a silicon chip to couple to a crystalline microresonator lying on its side^44^. However, this approach has obstacles, i.e., complex fabrication and alignment procedures, high losses, and an intricate design process. Photonic wire bonds (PWBs), additive 3D manufactured structures capable of guiding light in free-form configurations^45,46^, provide an alternative and more flexible coupling solution. Made out of VanCore A, a proprietary (Vanguard Automation GmbH) negative-tone photoresist similar to SU-8, these PWBs can arbitrarily be directly written onto the facets of fiber arrays (FAs) and even PICs, using a two-photon polymerization process. Recent work using PWBs to address the on-chip power bottleneck in thin film lithium niobate (TFLN) by enabling the direct-coupling of InP optical amplifiers to TFLN circuits^47^ has drawn attention to the versatility of these structures.

Here, we expand the use case of PWBs by evanescently coupling air-cladded variants to a MgF_2_ microresonator possessing a *Q*-factor around 1-billion and extinction ratio greater than 85% to address the challenge of crystalline microresonator coupling. Our PWB design results in PWB-FA facet losses as low as 0.85 dB/facet (at 1550 nm) and supports power handling of more than 400 mW. We use a directly coupled PWB-FA configuration to evanescently couple into a 4.92 mm diameter MgF_2_ microresonator and generate, for the first time, a soliton microcomb as well as a pure 14.2 GHz microwave beatnote tone with − 123 dBc/Hz (at 10 kHz offset) phase noise. Our results validate PWBs as flexible and robust coupling elements supporting evanescent coupling in addition to their more traditional usage in direct coupling. This demonstration opens the possibility of using PWBs with discrete material platforms, and offers a route towards passive integration of billion-*Q* crystalline microresonators with integrated photonics.

## Results

### PWB as a coupling element

Microresonator optical coupling primarily requires refractive index matching between the injected and circulating modes (k-vector matching) and benefits from a large evanescent field extent. While both properties exhibit sensitivity to PWB cross-section, the input-output arrangement and anchoring possess no similar constraints. In particular, use of a loopback structure for the PWB provides the most space-efficient geometry for evanescent coupling because it supports input and output ports written to the facets of a single eight-channel V-groove FA (Fig. 1a,b). With a production yield of 90%, the writing of these PWBs is both a repeatable and reliable process. The loopback’s power-handling capabilities are illustrated in Fig. 1c, which depicts thermo-reflectance imaging to identify regions with the highest optical power concentration and possible failure points^48^. At these limits, the PWBs fabricated in this work support maximum power levels up to 300 mW within the structure, i.e., 400 mW at the FA facet. Insertion loss (IL) at the FA facets depends on the PWB geometry at the interface. A cross-sectional diameter >14 µm at the anchoring point and a >100 µm long tapering down of the diameter to the coupling region minimizes losses. Evaluation of the end-to-end losses in our FA-PWB configuration, using polarization maintaining fibers with TE input (Fig. 1d) indicates that IL reaches approximately 1.7 dB (0.85 dB per facet). Taking into account coupling losses to the microresonator of 1.4 dB, the IL in the full FA-PWB-microresonator system lies around 3.1 dB. Additional PWB measurements, simulated properties, thermal cycling tests, and mechanical eigenfrequency analysis can be found in the Supplementary Information (SI).

The PWBs possess a nominal diameter of about 15 µm at the FA anchor point which then tapers down to 2 µm along the straight extension. Selection of this particular diameter results from the fact that the effective index ($n_\textrm{eff}$) for the fundamental TE mode can be engineered through the PWB geometry to match that of MgF_2_. Figure 1e illustrates this geometry-controlled phase-matching and indicates that larger PWB radii result in larger effective indices suitable for mode-matching with higher refractive index materials like SrF_2_, CaF_2_, and BaF_2_. The cross-sectional profile, however, need not be circular and can be designed to have an arbitrary elliptical profile. For example, a PWB thin in its major axis, but wide along its minor axis can enhance coupling via a large evanescent field while still supporting higher optical powers. After the straight extension, the structure undergoes a 90$^\circ$ bend with a 48 µm radius of curvature.

### PWBs for linear photonics

Figure 2a schematically illustrates the experimental setup used in characterizing the linear operating regime at low input optical power (< 10 mW). Input light from a tunable laser (Toptica CTL1550) is frequency-modulated by a 10 Hz triangular waveform to reveal a resonance mode spectrum. Using an external electro-optic modulator (EOM) to provide a time-frequency calibration on the oscilloscope, we evaluate the full-width half maxima (FWHM) of selected resonances and extract the associated quality factors. Figure 2b provides a 3D rendering of the FA-PWB-crystal system indicating all salient components and Fig. 2c shows a high-resolution photograph of a crystalline microresonator with diameter 4.92 mm, corresponding to a nominal free spectral range (FSR) of 14.2 GHz. Diamond turning defines the microresonator protrusion^32,33,49^ (Fig. 2c), which undergoes further polishing with diamond slurries to obtain a smooth surface and subsequently high *Q*-factor^50^. Both fabrication steps are compatible with automation and high-volume manufacturing.

After aligning the PWB with the equator of the microresonator protrusion (Fig. 2d) using a 3-axis precision stage, the gap between the two can be varied using a piezoelectric stage to provide undercoupled, critically coupled, and overcoupled states. In each configuration, it is possible to measure, as in Fig.  2e, the linewidth and quality factor of a particular resonance. Figure 2f shows the dependence of extinction ratio and resonance linewidth on the gap distance between the resonator and the PWB. Using the 3 MHz calibration sidebands from the EOM and normalizing the voltage at the photodetector output, the oscilloscope trace for the transmitted light across a resonance can be fit with a Lorentzian. This Lorentzian’s FWHM corresponds to the total microresonator linewidth, which can be described as $\kappa =\kappa _\textrm{0}+\kappa _\textrm{p}+\kappa _\textrm{ex}$^41,51^, and where $\kappa _\textrm{ex}$ is the external loss associated with the coupling, $\kappa _\textrm{p}$ is the parasitic loss, and $\kappa _\textrm{0}$ is the internal, intrinsic loss of the microresonator. The extracted linewidth of $\approx$ 240 kHz corresponds to a loaded *Q*-factor of $\sim$ 10^9^ at the input frequency of 193 THz (1550 nm), which is comparable to what has been measured in other MgF_2_ microresonators using other coupling mechanisms^44^. Additionally, the extinction ratio of approximately 85% measured in this critically coupled state indicates that coupling partially limits the linewidth because the transmission does not drop to zero on resonance, as would be expected in an ideal configuration. This non-ideal coupling can also be supplemented by a contribution from $\kappa _\textrm{p}$ arising either from light propagating in higher-order modes or a polarization mismatch relative to the resonator mode^51^.

### PWBs for nonlinear photonics

MgF$_2$ has a cubic nonlinearity and anomalous group velocity dispersion^52^, which allows for the generation of soliton frequency combs^1^. With $\sim$ 100 mW of optical power coupled into the microresonator, the resonances start to distort due to Kerr- and thermal-nonlinearities^52^. In order to reach a soliton state and lock into it, the experimental setup is modified to that shown in Fig. 3a. In particular, an EDFA is added in the signal path to augment the amount of power coupled to the microresonator and a set of ancillary components required for offset Pound–Drever–Hall (PDH)-locking are also included^53^. In addition to the oscilloscope used for monitoring resonances, an optical spectrum analyzer (OSA) and electrical spectrum analyzer (ESA) enable the observation of the full frequency comb in the single soliton state, as well as the associated beatnote, respectively.

With the EDFA, up to 400 mW of optical power appears at the FA-PWB interface, which means >300 mW resides within the thin coupling region of the PWB. The PWBs can sustain these power levels for hours of continuous operation, as well as repeated turning on/off of the system over days without burning, which speaks to the long-term stability of this coupling scheme. At this higher power level, soliton steps with finite length become visible in the transmitted and generated light of the resonance. By locking the input laser’s frequency within this step region using an offset PDH lock^53^, the compensation of chromatic dispersion by the nonlinear Kerr effect and the dissipation by the nonlinear gain, give rise to a dissipative soliton state^1^. When viewed on an OSA (Fig. 3b), the soliton takes the form of a frequency comb with a strong central line at the pump frequency and subsequent lines separated by the microresonator FSR = 14.2 GHz. The beatnote signal, which has a frequency corresponding to the FSR, is then measured using an ESA (R &S FSWP26A) as depicted in Fig. 3c. Low-noise microwave signals are of particular interest to precision metrology, navigation, telecom, and coherent radar^10,54^ and when offset PDH lock stabilized, crystalline microresonators can provide the stable frequency references required in these applications.

**Figure 4:**
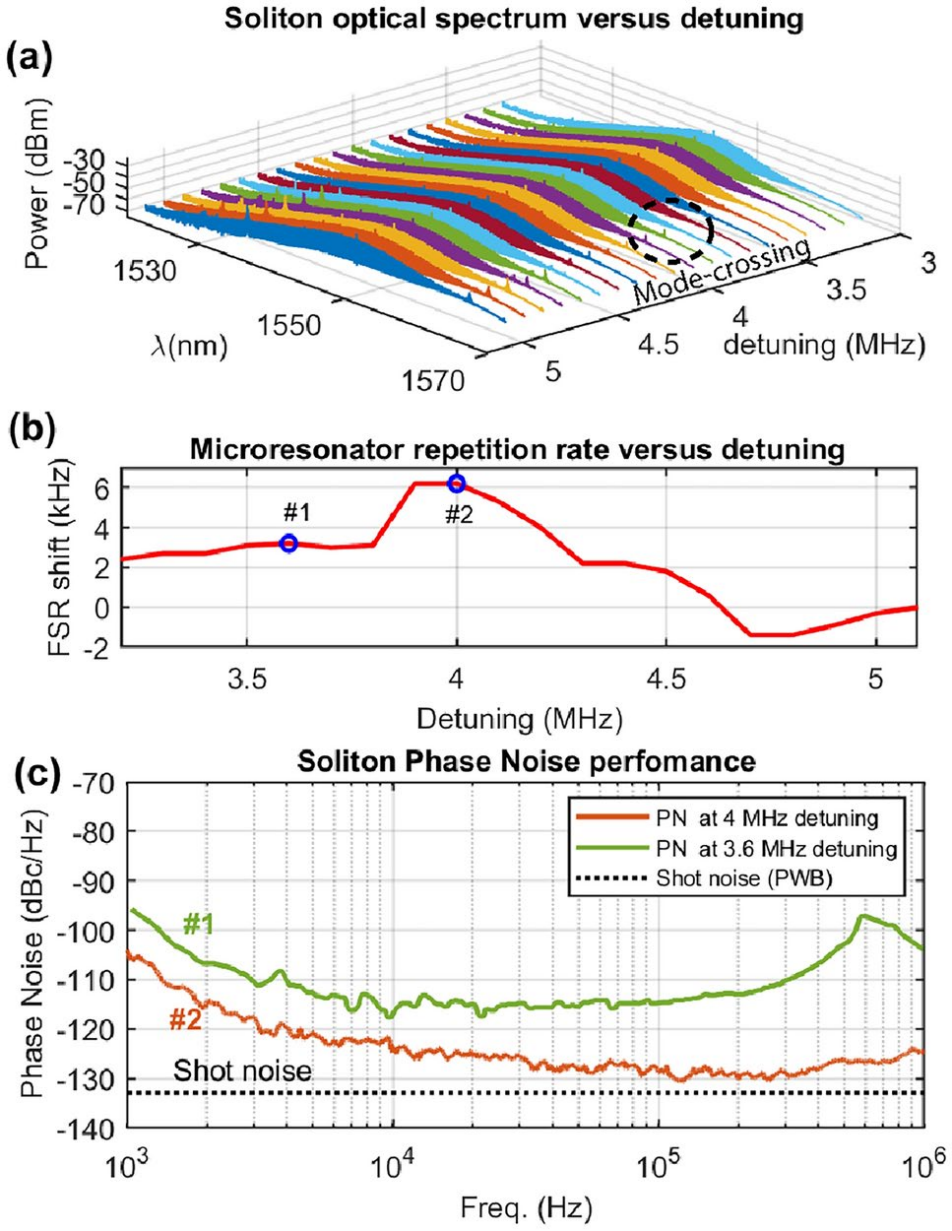
Quiet point hunting with PWBs. **(a)** Soliton spectra for a range of laser-resonance detunings from 3 to 5 MHz, where the modal crossings become visible after 4 MHz. **(b)** Visualization of the shift in microresonator repetition rate as a function of detuning. Point 1 indicates a normal operating point where the FSR varies linearly with detuning and Point 2 suggests a quiet point where the FSR versus detuning relation is effectively invariant. **(c)** Plot of the phase noise for the two points in (**b**) indicating around 10 dB noise suppression for point 2.

Within the soliton step, there are specific laser-resonance detunings where the transduction of input laser phase noise to FSR variation, or RF phase noise, is minimized (see SI). These states are often referred to as ‘quiet points’ and when the system is locked into such a configuration, the RF phase noise can be suppressed^10,55,56^. Searching for and identifying potential quiet points is guided by the fact that in this configuration, the FSR variation as a function of detuning is minimized^10^. After offset PDH locking into a soliton state, the detuning is stabilized and swept^53^. With each 100 kHz change, extraction of both the optical and electrical spectra reveals the existence and position of any modal crossings (Fig. 4a), the central frequency of the beatnote (Fig. 4b), and its associated phase noise (Fig. 4c). As avoided mode crossings have been linked to the evolution of FSR variation with detuning, the appearance of mode crossings in the spectra indicate a frequency regime promising the existence of quiet points^10^. The exact location can then be pinpointed and the noise suppression quantified, using phase noise measurements. The evolution of modal crossings and FSR shifts is shown in Fig. 4a,b and summarized in Fig. 4c, which provides an ESA measurement trace for a normal (Point 1) and a potential quiet point (Point 2) soliton. The latter possesses an average phase noise 10 dB lower than the former, which suggests a ‘quiet point’. Other detunings yield the same phase noise value as that exhibited at point 1 in the plot. Note that the phase noise in Fig. 4c increases beyond 100 kHz frequency offset and peaks at 600 kHz before decreasing again. This phenomenon can be linked to the PID parameters in the PDH locking that prioritize stability at lower offset frequencies, and which has been observed in other microcomb systems with feedback^29^.

## Conclusion and outlook

Crystalline microresonators, such as those based in MgF_2_, are ideally suited for nonlinear optics due to their potential for extremely high *Q*-factor, efficient GHz-range repetition rate solitons, and straightforward fabrication. However, as a discrete bulk element, leveraging this intrinsic advantage over other materials to generate solitons and low-noise microwave signals has hitherto required the use of sub-optimal coupling elements. PWBs challenge the status quo by offering a new means of coupling light into microresonators. In the last decade, PWBs have matured from a novel form of photonic chip interconnect^45,46^ to a commercially viable and scalable technology. Here we demonstrate the versatility of these free-form waveguides beyond standard point-to-point routing functionality between chips. PWBs can also serve as efficient evanescent coupling elements for generating solitons and low-noise microwave signals, with performance comparable to that found in traditional tapered fiber-microresonator or prism-microresonator systems. Not only can these PWBs provide low insertion loss and handle the optical powers necessitated by the nonlinear power threshold of MgF_2_, but they also hold the potential to support fully passive integration. In particular, we demonstrate effective and robust PWB coupling to a MgF_2_ microresonator with an extinction ratio of 85% and a quality factor $\sim$ 1 billion, as well as a soliton microcomb and pure (− 123 dBc/Hz at 10 kHz offset) microwave signal at a 14.2 GHz carrier.

Potentially one of the most exciting possibilities offered by PWBs is passive alignment to discrete photonic elements like crystals during the writing process, which drastically increases manufacturability. The results reported in this work come from PWBs written to V-groove FAs, which were then actively aligned to the microresonator protrusion to reach the optimal coupling position with high modal density and large extinction ratios. Such in situ positional monitoring is in fact not strictly necessary. Future iterations can envision writing the PWB directly from a PIC onto the microresonator protrusion at a pre-specified location (see SI). Neither component is displaced post factum and the need for active monitoring during alignment vanishes. Thanks to the robust optical coupling of PWBs, scalable PIC technologies can finally benefit from integration with high-performance crystalline microresonators. Moreover, PWBs need not be limited to microresonator-coupling: applications such as environmental sensing and light-driven soft robotics using fragile and bulky coupling components, like tapered fiber, profit from smaller more rugged PWB-based systems.

## Supplementary Information

Supplementary Information.

## Data Availability

All data needed to evaluate the conclusions in the paper are present in the paper and/or the Supplementary Materials.
