# Supplementary material for: Microresonator photonic wire bond integration for Kerr-microcomb generation
Source: Sci Rep. 2024 Nov 23;14:29054. doi: 10.1038/s41598-024-79945-4 (PMC11585607; doi:10.1038/s41598-024-79945-4)
Supplement: Supplementary file 1 — Supplementary Information. [file 41598_2024_79945_MOESM1_ESM.pdf]

Supplementary Information: Microresonator photonic wire bond integration for Kerr-microcomb generation

Alain Yuji Takabayashi[1†], Nikolay Pavlov[1†], Victoria Rosborough[2†], Galen Hoffman[3†], Lou Kanger[1], Farzad Mokhtari Koushyar[6], Taran Huffman[6], Mike Nelson[3], Charles Turner[4], Leif Johansson[2], Juergen Musolf[2], Henry Garrett[2], Thomas Liu[2], Gordon Morrison[2], Yanne Chembo[5], Brian Mattis[3], Thien-An Nguyen[6], Mackenzie Van Camp[4], Steven Eugene Turner[4], Maxim Karpov[1], John Jost[1*], and Zakary Burkley[4‡]

[1] *Enlightra, Rue de Lausanne 64, Renens, 1020, VD, Switzerland*
[2] *Freedom Photonics, 41 Aero Camino, Santa Barbara, 93117, CA, United States*
[3] *GXC, 10000 Metric Blvd., Austin, 78758, TX, United States*
[4] *FAST Labs™, BAE Systems, 130 Daniel Webster Hwy., Merrimack, 03054, NH, United States*
[5] *University of Maryland, 8279 Paint Branch Dr., College Park, 20742, MD, United States*
[6] *ORCA Computing, 10000 Metric Blvd., Austin, 78758, TX, United States*

1 Resonator Fabrication

Microresonator fabrication begins with cylindrical MgF_2 crystal rods that come in a variety of diameters. A 6 mm starting diameter crystal is fixed to a brass post with UV-curable glue. Next, as shown in Figure 1, a diamond tool lathe and single-point cut [1, 2], produce a 4.92 mm diameter microresonator protrusion with a 140 µm radius of curvature. This initial protrusion has the desired dimensions within micrometer precision, but possesses a rather rough surface, which necessitates a secondary polishing step. The crystal mounted on its brass post is inserted into a spindle and further polished using diamond grit slurries ranging from 10 µm particle size down to 250 nm and lint-free cloths [3].

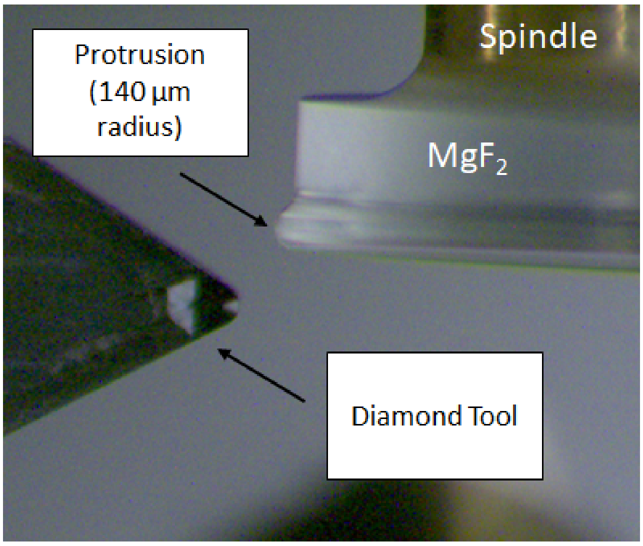

Figure 1: **Microresonator fabrication** Photo of the diamond tool used to cut the 140 µm radius of curvature protrusion in the mounted MgF_2 crystal.

† These authors contributed equally
* john.jost@enlightra.com
‡ zakary.burkley@baesystems.us

2 PWB Integration with Si_3N_4 PICs

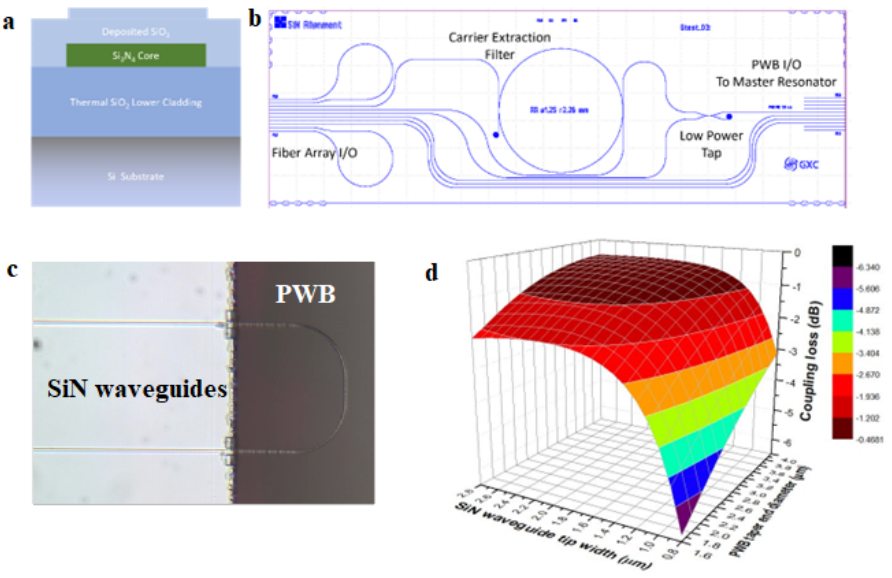

Figure 2: **PIC design and interface with PWB (a)** Cross-section of the SiN PIC indicating the various layers, which includes a Si substrate, a thermal oxide for the bottom cladding, a SiN core, and a deposited oxide for the top cladding. **(b)** Layout of a SiN PIC that can be used to interface with a fiber array and includes an on-chip carrier extraction filter to attenuate the strong pump line in the microresonator output. The right side of the chip also includes the facet upon which the PWB is drawn. **(c)** A PWB loopback written on a Si_3N_4 waveguide PIC. **d** Simulation of the coupling losses in the PIC-PWB interface as a function of dimensions for the SiN waveguide tip and PWB taper.

An ultra-low loss waveguide PIC, whose material platform cross-section is shown in Fig. 2(a), interfaces with the MgF_2 microresonator via a photonic wire bond (PWB) drawn on a polished chip facet. Typically, the platform includes TiN resistive heaters to provide thermal tuning to various components, but in this case, an external TEC provides the required tuning. Fig.2(b) shows a schematic of the PIC, which includes the fiber array input/output (I/O), PWB I/O, an on-chip resonator serving as the carrier extraction filter, and a low power tap (1:99) bypassing the on-chip filter.

The fiber array I/O, on the left side of the PIC includes waveguide mode converters exhibiting typical coupling losses of 2 dB, as well as waveguide loopback structures providing the optical feedback needed during the fiber array-to-chip attachment process. Design splits accommodating the various mode converter widths appear on the opposite side of the chip. Several additional paths between the fiber array and PWB I/O enable coupling schemes bypassing the on-chip filter entirely to promote easier initial testing. One output path from the microresonator includes an on-chip resonator serving as the carrier extraction filter, which provides 25 dB suppression of the central pump wavelength with minimal impact on the comb output. The low-power tap preceding the on-chip filter enables measurement of the full output spectrum.

A SONATA 1000 series system from Vanguard Automation draws PWB loopbacks to the Si_3N_4 PIC (Fig. 2(c)). This tool uses machine vision to align to the Si_3N_4 waveguide and uses two-photon polymerization to print arbitrary PWB paths, or in this case, a loopback structure. The PWB cross-section at the facet is 2-3 µm in diameter prior to the MgF_2 coupling region.

Simulations in Lumerical FDTD identify the coupling efficiency between the Si_3N_4 waveguides and PWBs with air cladding for various waveguide and PWB geometries (Fig. 2(d)). Here, simulations only take into account the interface between the PIC and PWB, thereby neglecting other components such as tapers. As shown in Fig. 2(d), a broad, low-coupling-loss region exists around a 2.1 µm wide Si_3N_4 waveguide and a 4 µm wide PWB. The resonator module PIC includes mode converter splits with 1.8, 2.1, and 2.3 µm wide Si_3N_4 waveguides. Freedom Photonics considered PWB geometries without a circular cross-section, and ultimately used a 5 x 3 µm elliptical profile because simulations indicated superior coupling to the elliptical Si_3N_4 waveguide mode.

Testing continued with PWBs written onto a fiber array-coupled SiN PIC, and subsequently aligned to a microresonator as part of a larger integrated subsystem (SI Fig. 3). The details and characterization of this integration can be found in the SI. This packaged device underwent full vibration testing according to the MIL-

STD-883G, which exposes devices to the potentially deleterious physical perturbations seen in military and space environments. The PWBs passed this mechanical test unscathed and maintained the desired alignment with the crystal to support nominal operation. Together with their thermal resilience at high optical powers, and integration in a PIC-based packaged assembly promising favorable Size Weight and Power (SWaP) metrics and demonstrated environmental ruggedness, PWB-based approaches offer versatile and robust optical coupling solutions.

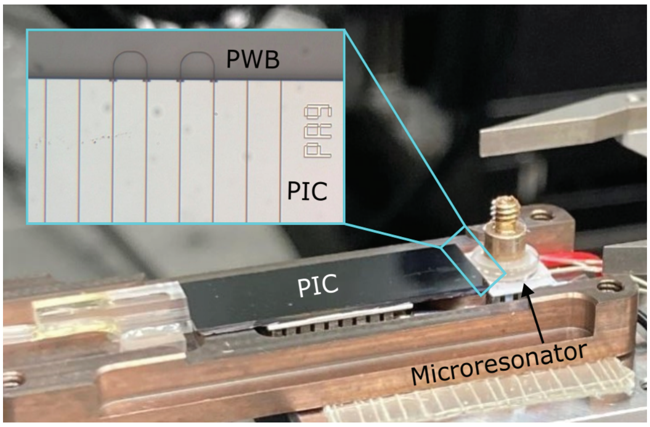

Figure 3: Photograph of a prototype assembly with PWBs written onto a fiber-coupled SiN PIC, (see inset) and aligned to a microresonator, within a package containing thermal control.

3 Material Properties and Testing of PWBs

The PWBs consist of an SU-8 photoresist provided by Vanguard Photonics. Simulation and evaluation of the developed and cured SU-8 material required information regarding the following mechanical properties:

- Mass Density
- Elastic or Young's Modulus
- Poisson's Ratio
- Yield Strength
- Ultimate Tensile Strength (UTS)
- Coefficient of Thermal Expansion (CTE)

Test structures included rectanglular, cantilever, and pull test devices and tests included a nanoindentation test, tensile test, flexural test, and coefficient of thermal conductivity test. Table 1 summarizes the measured material properties for the VanCore A photoresist material, which was polymerized using the same processing conditions as the final PWB loops. Numbers highlighted in green represent values utilized in COMSOL Multiphysics for the mechanical and multiphysics simulations.

Table 1: **SU-8 test results** Experimentally determined material properties for the cured SU-8 epoxy photoresist.

Material Properties Test Results

	**Displacement Rate [nm/s]**					
**Property**	0.1	1.0	10.0	100.0	1'000.0	10'000.0
Density [kg/m^3]	1190					
Material Hardness [MPa]			387	441	454	510
Tensile Yield Strength [MPa]	71.3	88.7	96.2			
Flexural Yield Strength [MPa]				74.9	79.9	
Ultimate Tensile Strength [MPa]	437.2	427.1	533.6			
Young's Modulus [GPa] - Nanoindentation			4.7	4.9	4.8	5.2
Young's Modulus [GPa] - Tensile Test	3.9	4.2	4.6			
Flexutral Elastic Modulus [GPa]				4.6	4.2	
Poisson's Ratio	0.5					
Coefficient of Thermal Expansion [ppm/K]	96.3					

Further investigation of the PWB as a suitable coupling element includes reliability testing over varying ambient temperatures and subject to various mechanical stresses. Testing the variation in output power over a temperature range from -40° to +85° C (Fig. 4) revealed an impressively low 0.3 dB peak-to-peak variation,

indicating that these PWBs not only support high optical powers but that they can also do so in drastically different environments.

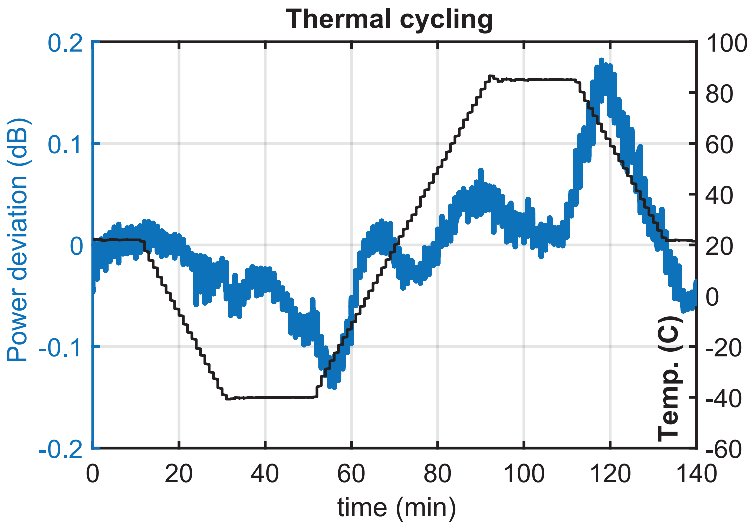

Figure 4: Plot of power fluctuations in the PWB during temperature cycling spanning -40° to 85° C range.

4 PWB Mechanical and Multiphysics Simulations

The novel approach of using PWBs as coupling structures necessitated a thorough understanding of their mechanical and thermal properties. Consequently, mechanical and multiphysics simulations looked at how the PWBs respond to various temperature extremes, temperature cycles, and vibration excitation. Mechanical simulations showing the material's ability to withstand mechanical vibration and shock not only allayed concerns regarding the presumed fragility of PWBs due to their small size, but thermal simulations also indicated a resilience to elevated temperatures. A relative correlation of these computational results with experimental data corroborates the assertion that PWBs exhibit remarkable robustness to various physical stresses.

The PWB geometry implemented in the Solidworks 3D Mechanical CAD System featured a waveguide pitch of 127 µm, a major diameter of 15 µm entering the tapered section, a taper length of 210 µm, a nominal diameter of 2 µm, a bending radius of 48 µm, and a total length of approximately 602 µm. Material properties came from the test results summarized in Table 1 and the ambient conditions consisted of air at 25°C and standard pressure at sea-level. Boundary conditions included a surface excitation applied to the interface between the waveguide and PWB, i.e., the 15 µm circular surface mating PIC Waveguides and PWB anchoring faces). Excitations directed perpendicularly to the propagation direction of light within the PWB constituted the worst-case mechanical perturbations.

Defining meshing parameters and verifying the mesh quality ensured an optimal eigenfrequency analysis. Results of this simulation show that the first eigenfrequency of the PWB occurs at approximately 73 kHz, which is significantly higher than the upper limit for the excitation frequency of 3 kHz. The high fundamental frequency of the PWB comes from the high elastic modulus of the cured SU-8 epoxy photoresist (elastic modulus = 4.7 GPa) and its extremely small weight (34.6 µg). This combination results in an extraordinarily high strength–to–weight ratio for the PWB and a resistance to bending.

Using the same environmental settings, material properties, and the solution data from the eigenfrequency analysis enabled a random vibration analysis to identify the maximum resonant RMS acceleration at the first eigenfrequency of 73 kHz. Although well outside the random vibration input frequency range, this resonant frequency allows for an estimation of the "worst-case" behavior of the PWB. In particular, the results indicate how displacement affects the mechanical coupling of the PWB to the optical resonator and identifies the resulting stress in the PWB. This information informed future designs so as to best ensure that the mechanical resonance does not damage the PWB.

The simulation uses a shifted PSD curve for the random vibration with a bandwidth center at 73054 Hz, (minimum RMS frequency = 72104 Hz/maximum RMS frequency = 74004 Hz), but whose total vibratory energy remains the same. This random vibration analysis identified a maximum RMS acceleration of +/- 74.16 m/s^2 in the vertical direction, which equates to an acceleration of 7.6 times the force of gravity (7.6 G).

5 Quiet Point Theory

In our work, the Kerr solitons are generated using a crystalline whispering-gallery mode disk-resonator with main radius a. The continuous-wave pump laser has a power P_{L} and angular frequency ω_{L}. Within the pumped-mode family, the eigenmodes are unambiguously labeled with their reduced azimuthal eigenumber l where $l = 0$ is the mode closest to the laser frequency, while the sidemodes are expanded as $l = \pm 1, \pm 2, \pm 3, \cdots$.

The pumped-mode family is characterized by an angular free-spectral range (FSR) Ω_{R}. Kerr comb generation can be modelled using a generalized Lugiato-Lefever equation [4, 5, 6]. The quiet point operation involves two eigenmode families A and B, and the generalized Lugiato-Lefever equations ruling the dynamics of the intracavity in the family A is [7]

$$\begin{aligned}\frac{\partial \mathcal{A}}{\partial t} &= -\kappa \mathcal{A} + i[\sigma + \rho \delta\Omega]\mathcal{A} + \frac{D_2}{2}\frac{\partial^2 \mathcal{A}}{\partial \theta^2} \\ &\quad + ig\tau_{\mathrm{R}} D_1 \mathcal{A} \frac{\partial |\mathcal{A}|^2}{\partial \theta} \\ &\quad + ig\,|\mathcal{A}|^2 \mathcal{A} + \sqrt{2\eta\kappa}\,\sqrt{\Phi} + iG\mathcal{B} \end{aligned} \tag{1}$$

where $\mathcal{B}$ is the eigenmode family B, σ corresponds to the detuning between the laser and the pumped resonance frequency in family A, τ_{R} is the Raman shock time, $g = n_2 c\hbar\omega_0^2 / n_g^2 V_{\mathrm{eff}}$ is the Kerr nonlinear parameter, $\Phi = P_{\mathrm{L}}/\hbar\omega_{\mathrm{L}}$ is the pump photon flux, η is the coupling efficiency (ratio between extrinsic and total losses), ρ is a resonator-dependent transduction coefficient, G is the coupling coefficient between two mode families, and Ω_{S} is the soliton repetition rate.

The fluctuations of the soliton repetition are such that

$$\delta\Omega = \Omega_{\mathrm{Raman}} + \Omega_{\mathrm{Disp}}\,, \tag{2}$$

where Ω_{Raman} and Ω_{Disp} are the the frequency-shift induced by the Raman and dispersive wave, respectively. Quiet point operation is achieved when $\delta\Omega = 0$, that is, when both frequency shifts cancel each other [7].

Funding

This research was supported by DARPA's GRYPHON program under contract no. HR0011-22-C-0039.

Declarations

The views, opinions, and/or findings contained in this article/presentation are those of the author(s)/presenter(s) and should not be interpreted as representing the official views or policies of the Department of Defense or the U.S. Government. Distribution Statement "A" (Approved for Public Release, Distribution Unlimited).

References

[1] Fujii, S. *et al.* Octave-wide phase-matched four-wave mixing in dispersion-engineered crystalline microresonators. *Opt. Lett.* **44**, 3146–3149 (2019).

[2] Min'kov, K. N. *et al.* Fabrication of high-q crystalline whispering gallery mode microcavities using single-point diamond turning. *J. Opt. Technol.* **88**, 348–353 (2021).

[3] Qu, Z. *et al.* Fabrication of an ultra-high quality mgf2 micro-resonator for a single soliton comb generation. *Opt. Express* **31**, 3005–3016 (2023).

[4] Matsko, A. B. *et al.* Mode-locked Kerr frequency combs. *Optics Letters* **36**, 2845–2847 (2011). URL `https://opg.optica.org/ol/abstract.cfm?uri=ol-36-15-2845`. Publisher: Optica Publishing Group.

[5] Chembo, Y. K. & Menyuk, C. R. Spatiotemporal Lugiato-Lefever formalism for Kerr-comb generation in whispering-gallery-mode resonators. *Physical Review A* **87**, 053852 (2013). URL `https://link.aps.org/doi/10.1103/PhysRevA.87.053852`. Publisher: American Physical Society.

[6] Coen, S., Randle, H. G., Sylvestre, T. & Erkintalo, M. Modeling of octave-spanning Kerr frequency combs using a generalized mean-field Lugiato–Lefever model. *Optics Letters* **38**, 37–39 (2013). URL `https://opg.optica.org/ol/abstract.cfm?uri=ol-38-1-37`. Publisher: Optica Publishing Group.

[7] Yi, X. *et al.* Single-mode dispersive waves and soliton microcomb dynamics. *Nature Communications* **8**, 14869 (2017).
